# A multi-step approach to developing a health system evaluation framework for community-based health care

**DOI:** 10.1186/s12913-022-08241-6

**Published:** 2022-07-09

**Authors:** Natalie C. Ludlow, Jill de Grood, Connie Yang, Sydney Murphy, Shannon Berg, Rick Leischner, Kerry A. McBrien, Maria J. Santana, Myles Leslie, Fiona Clement, Monica Cepoiu-Martin, William A. Ghali, Deirdre McCaughey

**Affiliations:** 1 Department of Family Medicine, Cumming School of Medicine, University of Calgary, Calgary, AB Canada; 2 W21C Research and Innovation Centre, Cumming School of Medicine, University of Calgary, Calgary, AB Canada; 3 Department of Human Centered Design & Engineering, University of Washington, Seattle, USA; 4 Faculty of Law, University of Calgary, Calgary, AB Canada; 5 Department of Health, Government of Alberta, Edmonton, AB Canada; 6 Departments of Family Medicine and Community Health Sciences, University of Calgary, Calgary, AB Canada; 7 Departments of Pediatrics and Community Health Sciences, University of Calgary, Calgary, AB Canada; 8 School of Public Policy and Department of Community Health Sciences, Cumming School of Medicine, University of Calgary, Calgary, AB Canada; 9 Department of Community Health Sciences, Cumming School of Medicine, University of Calgary, Calgary, AB Canada; 10 Cumming School of Medicine, McCaig Institute for Bone and Joint Health, University of Calgary, Calgary, AB Canada; 11 Office of the Vice-President (Research), University of Calgary, Calgary, AB Canada

**Keywords:** Community-based Health Care, Health System Research, Modified Delphi, Evaluation Framework, Indicator Development

## Abstract

**Background:**

Community-based health care (CBHC) is a shift towards healthcare integration and community services closer to home. Variation in system approaches harkens the need for a conceptual framework to evaluate outcomes and impacts. We set out to develop a CBHC-specific evaluation framework in the context of a provincial ministry of health planning process in Canada.

**Methods:**

A multi-step approach was used to develop the CBHC evaluation framework. Modified Delphi informed conceptualization and prioritization of indicators. Formative research identified evaluation framework elements (triple aim, global measures, and impact), health system levels (tiers), and potential CBHC indicators (*n* = 461). Two Delphi rounds were held. Round 1, panelists independently ranked indicators on CBHC relevance and health system tiering. Results were analyzed by coding agreement/disagreement frequency and central tendency measures. Round 2, a consensus meeting was used to discuss disagreement, identify Tier 1 indicators and concepts, and define indicators not relevant to CBHC (Tier 4). Post-Delphi, indicators and concepts were refined, Tier 1 concepts mapped to the evaluation framework, and indicator narratives developed. Three stakeholder consultations (scientific, government, and public/patient communities) were held for endorsement and recommendation.

**Results:**

Round 1 Delphi results showed agreement for 300 and disagreement for 161 indicators. Round 2 consensus resulted in 103 top tier indicators (Tier 1 = 19, Tier 2 = 84), 358 bottom Tier 3 and 4 indicators, non-CBHC measure definitions, and eight Tier 1 indicator concepts—Mortality/Suicide; Quality of Life, and Patient Reported Outcome Measures; Global Patient Reported Experience Measures; Cost of Care, Access to Integrated Primary Care; Avoidable Emergency Department Use; Avoidable Hospitalization; and E-health Penetration. Post Delphi results refined Tier 3 (*n* = 289) and 4 (*n* = 69) indicators, and identified 18 Tier 2 and 3 concepts. When mapped to the evaluation framework, Tier 1 concepts showed full coverage across the elements. ‘Indicator narratives’ depicted systemness and integration for evaluating CBHC. Stakeholder consultations affirmed endorsement of the approach and evaluation framework; refined concepts; and provided key considerations to further operationalize and contextualize indicators, and evaluate CBHC as a health system approach.

**Conclusions:**

This research produced a novel evaluation framework to conceptualize and evaluate CBHC initiatives. The evaluation framework revealed the importance of a health system approach for evaluating CBHC.

## Background

Health systems everywhere are increasingly recognizing that high-performing systems need more than just hospitals. Community-based health care (CBHC) is a paradigm approach that emphasizes the decentralization of care away from acute and institutional settings towards delivering health and social services closer to home [1, 2]. The CBHC paradigm covers a broad range of integrated health and social services within the community in response to meeting the healthcare needs of people [2, 3]. In countries with growing numbers of seniors living in community settings, the CBHC paradigm allows people to age in place along the care continuum, avoid unnecessary hospital stays, and increase access to resources where they are needed most [1, 4]. In Canada, high rates of emergency department visits for conditions that could be managed within the primary care setting; varying access to first line health services; and building communities where the healthy choice is the easy choice and people can age in place, are examples of healthcare needs where the CBHC paradigm can respond [2]. Yet, implementing CBHC as part of the wider healthcare system, will require complex integration of health, social, and community services, as well as jurisdictional and health reforms [2, 5, 6].

While the need for CBHC is widely recognized and many health systems are implementing some form of CBHC, there is considerable variation in the structure, function, and implementation. Specifically, these variations have been shown to differ by care setting, geographic region, disease/condition targeted, and general approach to addressing patient needs in communities [7–9]. Additionally, CBHC requires a shift in thinking, transforming services to meet population needs more locally and reducing fragmentation in service delivery [10]. As such, the complexity of CBHC requires clear conceptualization and evaluation for successful strategic direction and policy planning.

The variation in approaches to defining, designing, and implementing CBHC, harkens a need to create a conceptual evaluation framework for CBHC. Alongside this need for a well-defined CBHC evaluation framework, there is a need for robust evaluation criteria and associated quality indicators for CBHC. Recognizing these gaps, we undertook a multi-step approach to develop a CBHC-specific evaluation framework in the context of a provincial CBHC planning process. In collaboration with the provincial Ministry of Health in Alberta, a Canadian province, a CBHC evaluation framework was developed and applied to a set of community-based programs to produce a consolidated set of indicators relevant to CBHC. The general approach taken and resulting evaluation framework and indicator set are likely to be of relevance and interest to health systems globally, as they contemplate and develop their own integrated CBHC strategies.

## Methods

### Developing a community based health care evaluation framework

**Fig. 1:**
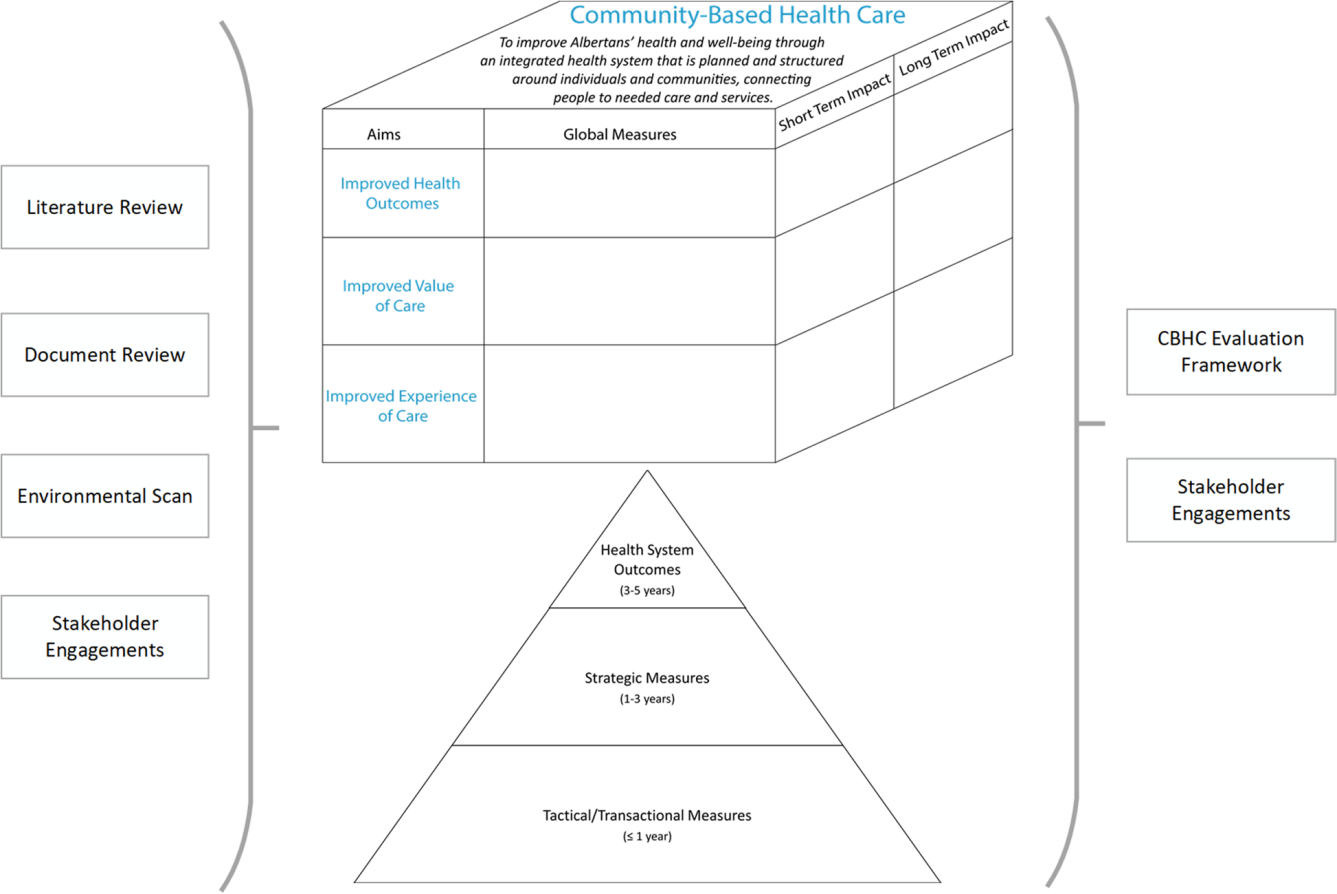
CBHC evaluation framework development process

This research was commissioned by Alberta’s Ministry of Health as a central part of policy work to support future decision making about implementing a system-level CBHC in the province. The intention of this research was to present a framework to facilitate the continuous evaluation of a system-level CBHC initiative. To create the desired evaluation framework, a multi-step approach was conducted: a) a literature review of existing CBHC frameworks and indicators; b) a review of the Ministry of Health’s internal documents and existing indicators for measuring CBHC; c) identification of an extensive listing of potential candidate indicators; d) a modified Delphi process to determine the candidate indicators that should be included in the evaluation framework; and e) consultation with stakeholders to endorse and capture feedback for next steps (Fig. 1). This manuscript focuses on the last two steps of the approach in the CBHC evaluation framework development. Nevertheless, to give context of the process, we will present a brief overview of the initial steps (a-c above) here as well.

The Ministry of Health identified several system-level programs that, together, comprised a core set of CBHC initiatives: 1) a bundle primary care reforms; 2) information technology tools for care integration, 3) a mental health action plan; 4) a community paramedicine program; 5) home and community care enhancements; 6) a dementia action plan; 7) enhanced continuing care; 8) organized chronic disease prevention/management strategies, and 9) a healthy communities plan. Document review and consultation with the program stakeholders laid the foundation for the CBHC-aligned initiatives. These activities provisioned current indicators already in use by the system-level programs, and a system-level performance measurement framework gave insight into the different levels of the province’s health system.

A three-dimensional matrix was then drafted to depict: 1) the Ministry of Health’s adapted three improvement domains of the Triple Aim [11]—improved health outcomes, value of care, and experience of care; 2) global CBHC measures; and 3) the measurement time frame of indicator relevance (i.e., time to show impact). Program area stakeholders mapped current program indicators to this matrix. An environmental scan of other Canadian initiatives and a narrative literature review were also conducted to identify evaluation frameworks applicable to the CBHC program areas and the Ministry of Health’s CBHC initiative as a whole. The literature review used a comprehensive search strategy that retrieved 2942 articles. Two reviewers screened and extracted information, followed by a team review and synthesis of the results. These initial steps led to a basket of potential indicators (*n* = 461) that were further refined through the modified Delphi process.

### Modified Delphi process

An expert review process was undertaken to assess each of the candidate indicators identified for their relevance to CBHC, and for their appropriateness for inclusion in the final evaluation framework. The modified Delphi technique is commonly used to achieve consensus and identify specific indicators in health service research (e.g., [12–15]). For this study, nine content experts from the University of Calgary were invited to participate as panelists. Eight were available to participate (88.9%). Panelists ranged in age and gender, and at various stages in their research career. Panelists had health system/services expertise in healthcare management, health policy, primary care, patient-centred care, quality of life, elder care, population health, health systems evaluation, and quality improvement.

Panelists were oriented to the purpose of the CBHC evaluation framework as a Ministry of Health system-level initiative with core program areas; presented with inclusion/exclusion criteria for selection of indicators, and provided an explanation of the rating process. The inclusion criteria (Table 1) were developed based on a performance measurement framework that was in use by the Ministry of Health. This performance measurement framework used different tiers of importance for considering health system indicators —Tier 1: high level health system outcomes, Tier 2: strategic program-level measures, and Tier 3: unit-level measures or tactical and transactional measures. A fourth category was included to allow Delphi panelists to categorize indicators that were either not specific enough in their descriptions or felt to not be CBHC-related.

**Table 1:** Inclusion criteria for ranking indicators into Tiers

Tiers	Definition for ranking
1. Health System Outcomes	High-level measures
	• Resonate with the public
	• Potential integration across program areas and inclusion of community resources
	• Ability to be benchmarked nationally/internationally
	• Targets typically achievable in 3–5 years
2. Strategic Measures	Program-level measures
	• Tightly linked to CBHC program areas
	• Focus on proven drivers of health system outcome measures
	• Mostly focus on proven structure and process
	• Disease pathways that have the most impact on the health of the population and the health system (cost/resources)
	• Targets typically achievable in the first 3 years
3. Tactical/Transactional Measures	CBHC Relevant, but not Tier 1 or 2
	• Linked to CBHC program areas, but are not health system outcomes or strategic measures
	• May be focused on individual program level
4. Not CBHC—Specific	Does not seem to be related to CBHC or any of the CBHC program areas

The panelist rating process consisted of two rounds of sequential review and revision. In the first round, panelists independently ranked the full list of indicators according to the inclusion criteria above. Results were merged into a master spreadsheet and coded for analysis. Simple descriptive analyses were used to determine agreement of indicator tier rankings across reviewers, with a focus on determining disagreement of indicator ranking assignments into the top (Tier 1 & 2) vs. bottom (Tier 3 and 4) tiers (Table 2). Indicator consensus was defined as 75% or more agreement—highest and lowest rating removed—in keeping with typical modified Delphi indicator rating processes [16]. Additional descriptive statistical analysis and measures of central tendency were reported to further characterize the results.

**Table 2:** Codes for indicator ranking analysis

Code	Code Description
Code A	Disagreement for Tier 1 or 2 vs. 3 or 4
Code B	Disagreement only for Tier 1 vs. 2
Code C	Disagreement only for Tier 3 vs. 4
Code D	Agreement for Tier 1
Code E	Agreement for Tier 2
Code F	Agreement for Tier 3
Code G	Agreement for Tier 4

In the second round, panelists attended an in-person consensus meeting to discuss disagreement and further deliberate the parameters of indicator placement in the tiered system. Median scores and standard deviation (SD ± 1) from the code analysis were used to facilitate consensus building discussions on indicators where disagreements were found. Indicators were tiered and thematic groups were identified. Thematic concepts were grouped as indicators measuring similar CBHC domains (e.g., of relevance to primary care reforms: access to primary care, time-to-appointment with primary care physician, and regular appointments with primary care physician). Panelists focused on defining Tier 1 indicators and thematic indicator concepts, as well as determining indicators that were not CBHC relevant, while conceptualizing generalizations of what constituted the remaining tiers. The Tier 1 emphasis aligned with the Ministry of Health’s goal of developing a CBHC evaluation framework at the system/provincial level. Panelist discussions were used during post-Delphi rounds to further refine the indicators into their respective tiers and thematic grouping of the concepts.

### CBHC evaluation framework and stakeholder consultation

Using the results from the modified Delphi, tiering was refined and lower tiered indicators were grouped into concepts. The proposed three-dimensional matrix was used to show system level health outcome measures (Tier 1) in relation to the Ministry of Health’s adaptation of the Triple Aim [11], and the length of time (short or long term) expected to see impact. Tier 1 Delphi results were plotted to check for any possible gaps. The evaluation framework presented here focuses primarily on the highest tiers indicators. However, all indicators selected in Tiers 1, 2, and 3, were mapped to their respective tier. Further, some examples of “indicator narratives” were developed to demonstrate how indicators relate to one another, across tiers, in this integrated evaluation framework.

Stakeholder consultations with the Ministry of Health occurred throughout the development process. Once development was complete, the Ministry of Health co-facilitated three stakeholder consultations to present the evaluation framework for endorsement and feedback. The first meeting was an in-person meeting with scientific experts. The second and third meetings were broader stakeholder engagements (e.g., with Ministry of Health staff, health service leaders, and patients) held in two of the province’s largest urban centres. The invited stakeholders included: knowledge experts of the core CBHC program area initiatives, stakeholders with broad provincial economic and strategic views, and stakeholders with lived experience of the health system (patient perspective). Having broad stakeholder engagement was important to check for acceptance in the approach and the results of work. The information gathered from these meetings was triangulated and used to inform caveats, recommendations, and next steps for provincial scale-up of CBHC and the final proposed evaluation framework.

### Ethical considerations

This study falls under quality assurance/program evaluation. The Conjoint Health Research Ethics Board (CHREB) at the Cumming School of Medicine, University of Calgary waived the requirement for research ethics review as per the TriCouncil Policy Statement 2014—Chapter 2, Article 2.5. All methods were carried out in accordance with relevant guidelines and regulations.

## Results

### Identification and review of candidate indicators

**Fig. 2:**
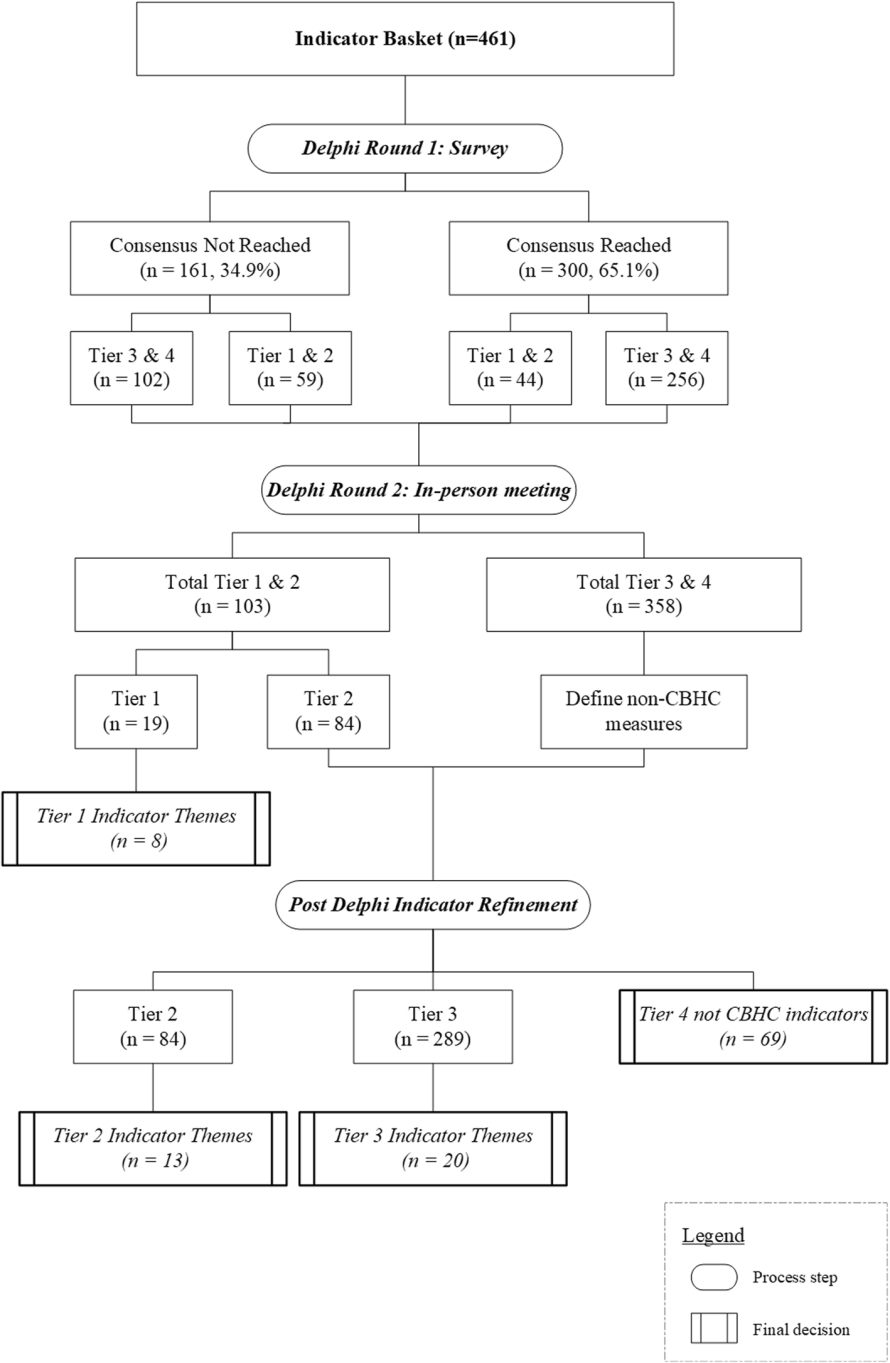
Indicator development flow diagram

A multi-source search process for candidate indicators yielded a total of 461 unique candidate indicators (after sorting and reconciliation of duplicate indicator concepts). Figure 2 presents the results of the indicator ranking process. In round one, there was agreement on 300 of the 461 indicators, with some of the indicators assigned to the upper tiers (Tier 1 & 2; *n* = 44) or lower tiers (Tier 3 & 4; *n* = 256). The remaining 161 indicators (35%) had disagreement in ratings and were brought forward for the face-to-face meeting (round 2).

The face-to-face meeting (second round review) brought agreement on indicator tier assignments for the 161 indicators where there was disagreement in round 1. Among these, 59 indicators were reassigned with agreement to the upper tiers (Tiers 1 & 2) and 102 were reassigned with agreement to the lower tiers (Tiers 3 & 4). This led to a total of 103 indicators assigned to the upper tiers. Next, the panel began differentiating between Tier 1 and Tier 2 indicators. In this step, panelists used the inclusion criteria and results from round 1Tier 1 and 2 indicators to decide upon what constituted Tier 1 indicators—i.e., those that reflect meaningful health system outcomes. From this rating step, consensus was achieved by the panel; 19 indicators were identified as Tier 1 indicators and 84 indicators as Tier 2.

Panelists then proceeded to group Tier 1 indicators into the following eight concepts: 1) Mortality/Suicide; 2) Quality of Life and Patient Reported Outcome Measures (QoL/PROMs); 3) Global Patient Reported Experience Measures (PREMs); 4) Cost of Care; 5) Access to Integrated Primary Care; 6) Avoidable Emergency Department (ED) Use; 7) Avoidable Hospitalization; and 8) E-health Penetration. The Delphi panel determined that indicators in the integrated primary health care and e-health penetration concepts are extremely important to CBHC implementation at the health system level (and thus were placed into Tier 1, even though they are not health outcomes, per se).

Once indicators in the upper tiers were reconciled, panelists spent time assigning the remaining indicators (not assigned to Tiers 1 or 2) to Tier 3 vs. Tier 4. Centrally, this involved determining which of the lower tier indicators were not relevant to CBHC. Among 358 indicators assigned to lower tiers through the two-step rating process, a total of 289 indicators were assigned, with consensus, to Tier 3 (tactical and transactional measures of relevance to CBHC). The remaining 69 indicators, meanwhile, were determined to be not relevant or related to CBHC (Tier 4).

Lastly, the panel considered groupings of Tier 2 and Tier 3 indicators that align with (i.e., connect to) the eight Tier 1 concepts listed earlier. This process also yielded additional concepts unique to Tier 2 and/or Tier 3 indicators. Table 3 presents the indicator concepts and their alignment with Tiers 1, 2, and 3 indicators.

**Table 3:** Core concepts identified within each tier from the modified Delphi review process

Concept	Tier 1	Tier 2	Tier 3
Mortality/Suicide	•	•	
Quality of Life/Patient Reported Outcome Measures	•	•	
Global Patient Reported Experience Measures	•	•	•
Cost of Care	•	•	•
Access to Integrated Primary Care	•	•	•
Avoidable Emergency Department (ED) Use	•	•	•
Avoidable Hospitalization	•	•	•
E-health Penetration	•		
Wait Times		•	
Geographical PCN Coverage		•	
Public Health		•	•
Morbidity/Incidence		•	•
Continuing Care Capacity		•	•
Effective Palliative Home Care		•	
Access to Home Care Services			•
Access to Social Services			•
Advance Care Planning			•
Community Paramedicine Capacity			•
Coordination of Care			•
Organizational Effectiveness			•
Patient Demographics			•
Patient Self-efficacy			•
Patient-centred Care			•
Provider Experience and Training			•
Quality of Care			•
Team-based Care			

### Interposing the Delphi panel results into the final CBHC evaluation framework

**Fig. 3:**
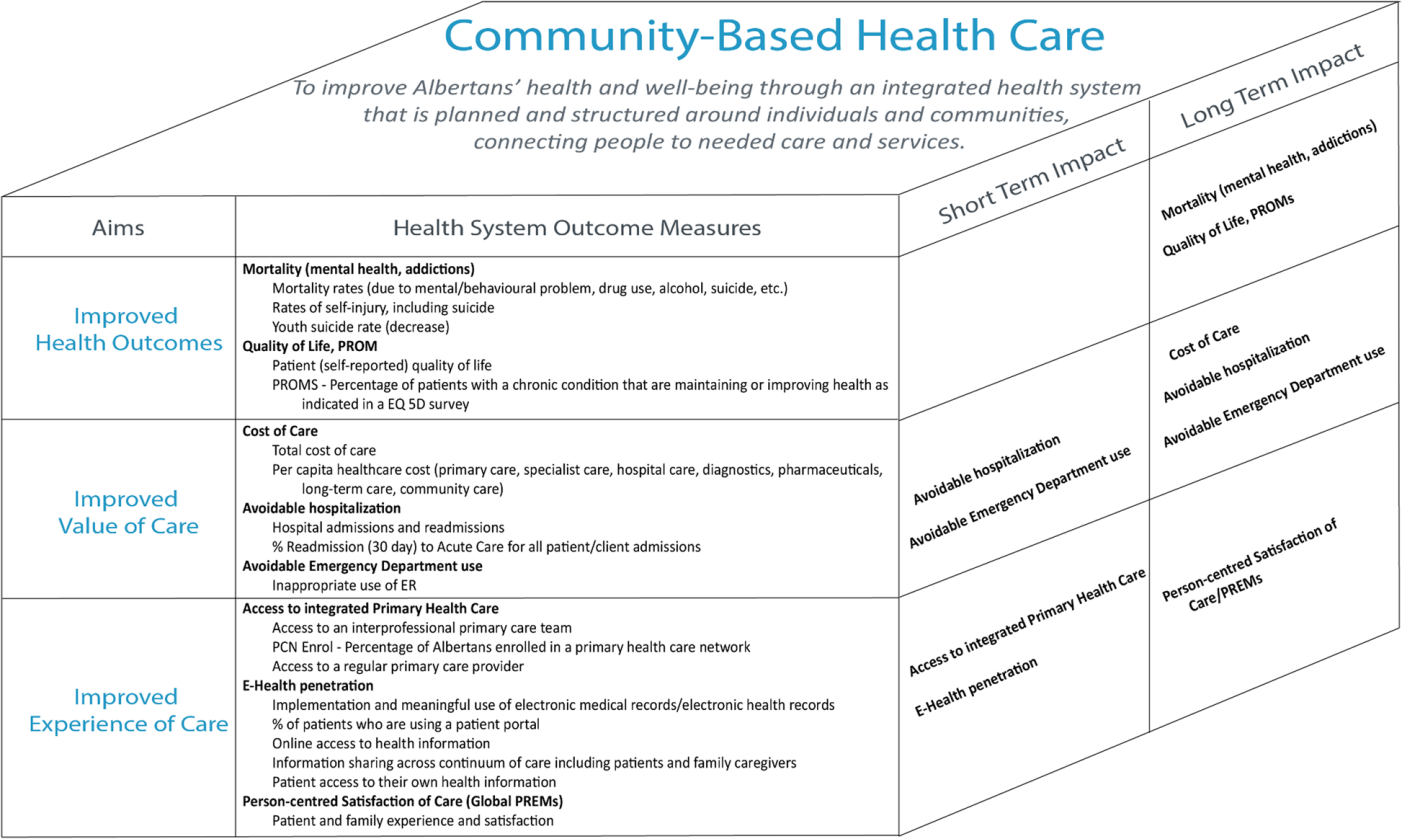
CBHC evaluation framework

The research team mapped the Tier 1 concepts and 19 respective indicators to the proposed three-dimensional CBHC evaluation framework (Fig. 3). When mapped, these concepts and indicators revealed comprehensive coverage across each of the adapted Triple Aim dimensions. As defined, these Tier 1 health system outcome measures are likely to show impact in the long term (3–5 years). Two indicator concepts—access to integrated primary health care and e-health penetration—are more structural/process measures and have potential to demonstrate positive changes and impact even sooner (≤ 3 years).

**Fig. 4:**
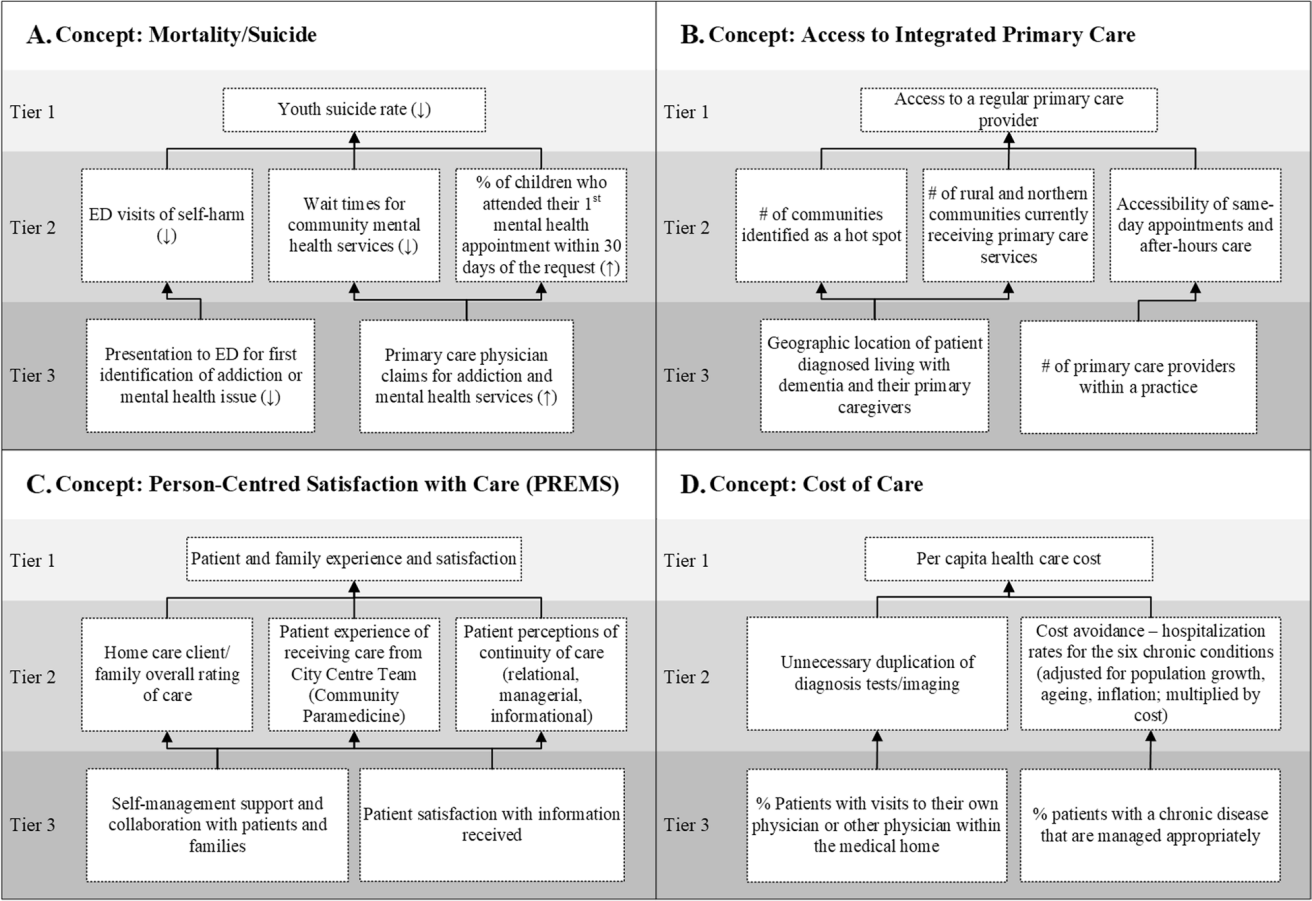
Examples of multi-tier indicator narratives around the concepts of: **A **Mortality/Suicide; **B **Access to Integrated Primary Care; **C **Person-centred Satisfaction with Care (PREMs); and **D **Cost of Care. Note: These are examples of narratives; additional indicators in Tiers 2 and 3 will likely also feed into the Tier 1: High-level Measures–Health Systems Outcomes

The proposed CBHC evaluation framework (Fig. 3) represents only the top tier, system-level concepts and indicators pertinent to the Ministry of Health’s CBHC initiative. To illustrate the additional tiers and respective indicators, a series of “indicator narratives” were developed. These narratives (Fig. 4) comprehensively depict how indicators across the different tiers are related to one another, and thus integrated (Fig. 4). Through these indicator narratives, vertical and horizontal integration of indicators at different levels within the health care system are identified. Thus, tactical/transactional concepts (Tier 3) map to concepts at the strategic level (Tier 2), and the strategic level indicators in turn map to concepts that are health system outcome (Tier 1) indicators. There is also a horizontal relationship in that two or more indicators together can be used to inform higher level system outcomes.

To elaborate on this explanation of indicator narratives, the integration of indicators for the concept Cost of Care is used as an example (see: Fig. 3, panel D). In this example, a Tier 3 indicator measuring ‘% patients with a chronic disease that are managed appropriately’ was mapped to the concept ‘Quality of Care.’ This indicator provides valuable information about chronic disease management, which can impact a Tier 2 indicator such as ‘cost avoidance: hospitalization rates for the 6 chronic conditions’, belonging to the ‘Cost of Care’ concept. Together, the two indicators provide a deeper understanding of strategic structures and processes in place across multiple program areas. In turn, this may further inform a Tier 1 indicator measuring ‘per capita health care cost’ at a system-wide level, mapped to the ‘Cost of Care’ concept, at the highest, health system outcomes level. The three indicators, while meaningful at different tiers, provide evaluators a comprehensive understanding of performance of a health care system in the cost of care domain, as it pertains to CBHC.

### Stakeholder consultations

Engagements revealed unanimous endorsement for the approach taken to develop the evaluation framework and agreement of the content and face validity of the framework elements. Stakeholders presented important feedback about the evaluation framework and four points from this feedback led to concept refinement that was included as recommendations/considerations in the final report to the Ministry of Health. First, it was noted that certain indicators assigned to Tier 4 (“not CBHC relevant”) were, nevertheless, felt to be important population health indicators, despite their being unrelated to the CBHC programs. Second, stakeholders asked how the evaluation framework can account for (and/or measure) the social determinants of health (i.e., stratifying to account for known social determinants). Third, stakeholders worried that the notion of “tiers” is hierarchical and may lead to judgement on the inherent value of indicators linked to tier assignments. Fourth, some stakeholders wondered whether there are indicators or measures that might transcend the tiers that were used for this evaluation framework exercise—i.e., higher-level measures of system integration, perhaps described as a ‘Tier 0’ or ‘meta-indicators’.

## Discussion

We have presented an in-depth multi-step approach used to develop a novel CBHC evaluation framework for Alberta’s Ministry of Health. The modified Delphi methodology informed the development and strategic prioritization of key indicators and associated concepts that align with CBHC initiatives. The evaluation framework and its associated indicator tiers map well to a strategic matrix based on the Triple Aim, thus affirming the strategic aims of the Ministry of Health. While the CBHC evaluation framework presents indicators and concepts at the highest systematic level, additional indicator narratives conceptually explain the relationships of indicator measures across the tiers. Further, stakeholder consultations endorsed the developed evaluation framework, while at the same time identifying important points that invoke broader considerations that would be needed for next steps to applying the framework. The general approach on the evaluation framework development will be of value to any readers involved in CBHC and/or health system improvement activities. Similarly, the resulting evaluation framework and indicator set are likely to be of use for evaluation of CBHC initiatives in any jurisdiction.

This research was informed and designed by current CBHC literature (peer and grey literature), Ministry of Health documentation and strategic frameworks, CBHC frameworks from other jurisdictions, and sentinel conceptual frameworks such as the triple aim [11, 17]. Importantly, there are few system-wide CBHC initiatives. Rather, initiatives tend to be targeted at a specific program or disease condition [2, 9]. The Triple Aim framework is widely used in health system/services research and is being applied in practice to inform evaluations of health system and program performance [18–20].

Although the CBHC evaluation framework was developed for a specific province, it transcends jurisdictions and is applicable to other health systems implementing CBHC. In Canada, provinces are implementing Community-based Primary Care models that focus on a specific setting or condition [3, 21]. The system-level CBHC evaluation framework presented here is informed by national and international literature on evaluation frameworks and candidate indicators. As well, it incorporates the local context for CBHC as a decentralized approach to health care delivery that transcends different health settings.

Stakeholders were continuously engaged throughout this work—an approach that enriched the development of this evaluation framework. Working closely with health system partners in the program areas gave insight into understanding the vision for CBHC, while also informing on current operational considerations for the evaluation framework’s development. Broader stakeholder consultation with knowledge expertise in the program areas, provincial health governance, and patient perspective led to important specific considerations. Further, prepublication oral presentations of this evaluation framework to international audiences sparked considerable interest and dialogue with international partners. From these engagements, important caveats were identified as necessary next steps to support the practical application of the CBHC evaluation framework in applied health system evaluations.

First, a key next step is the need for broader stakeholder engagement and input to better examine local and national contextual factors. There is little consensus in the literature on sets of measures for CBHC initiatives, due to the diversity of how CBHC is implemented [9, 22, 23]. Stakeholders at different levels of governance (local to regional, to provincial, to national) may have divergent perspectives on strategic direction for CBHC policy and planning. Broader consultations would inform the development of both tailored and generalizable concepts to for CBHC evaluation. While concepts may be generalizable, the constructs of indicators and targets for outcomes are likely to require consideration of local context, equity, and inclusivity. To do this, engagement with specific populations and communities are required (examples include but not exclusive to Indigenous, newcomer, LGBTQ2S + , and elderly and ageing populations). Stakeholder engagements from different levels of governance and with different communities can identify additional contextual factors that can facilitate or impede implementation, and ultimately inform improvements of the evaluation framework.

A second task is to operationalize the proposed set of indicators. Refinements to the indicators are needed and explicit indicator definitions must be created, with consideration of the underlying data sources and indicator methodology. The result of this task would be an indicator manual (of sorts), with roadmap for requisite data and methodology for constructing CBHC-specific indicators. While CBHC could transform health service delivery to serve population health needs closer to home [10], it is important to focus on measuring impact specific to CBHC initiatives. The inclusion of broad health indicators (e.g., disease incidence/prevalence) risks confounding attempts to measure (more specifically) the impact of CBHC.

A third consideration will be to explicitly account for social determinants of health in the CBHC evaluation framework. It is widely accepted that health is affected far more by social determinants (e.g., income, education, social status, occupation, ethnicity, location of residence) than it is by health services received or not received. Efforts to operationalize indicators, therefore, need to account for social determinants of health, because these have direct effects on key health outcomes and indicators. Any health indicator-based assessment of CBHC impact using the evaluation framework developed here would need to consider the potential modifying effects on CBHC impact of various social determinants. Analytically, this is likely best assessed by the conduct of indicator analyses, stratified on key social variables of greatest interest and importance (e.g., impact on Indigenous vs. non-Indigenous people, or impact across income strata). Such analyses would allow for explicit assessment of differential effects across sociodemographic and socioeconomic strata.

Lastly, there is a need to consider system integration (‘system-ness’) of CBHC programs. Integration of care is a concept with multiple dimensions of continuity such as relational continuity (e.g., longitudinal care relationships with providers), informational continuity (e.g., integration of accessible health information across the lifespan and care journey), and management continuity [24, 25]. The evaluation framework, as developed and presented, does not explicitly capture the system integration construct (i.e., measures that CBHC is being integrated within the health system). It does, however, implicitly capture concepts of continuity in individual indicators and their connection across the health system Tiers. For example, some key Tier 1 indicators capture relational continuity (e.g., indicators of integrated interdisciplinary primary care) and informational continuity (e.g., E-health penetration indicators). Management continuity, meanwhile, is reflected in some of the Tier 3 tactical and transactional indicators that relate to health system activity and integrated governance structures to support longitudinal/multi-system care. These embedded integration constructs notwithstanding, there may still be a need to conceptualize and incorporate a composite measure of system integration, perhaps drawing on combined measures of integration, as just discussed.

## Conclusions

In closing, this paper presented a multi-step process to developing an evaluation framework for CBHC. A key aspect of the work presented here is its interdisciplinarity and the longitudinal partnership with health system decision-makers and other health system stakeholders at all steps of framework development, refinement, and subsequent stakeholder engagement. The process undertaken in framework development is likely to be of value to health systems and health services researchers involved in the development of other types of system evaluation frameworks. The resulting CBHC evaluation framework presented here can be a resource for the Canadian province where the work was conducted, and it is likely to also be of interest to any other jurisdictions considering their own evaluation frameworks of community-based care programs.

## Data Availability

The datasets generated and/or analyzed during the current study are not publicly available due to continuing refinement of the evaluation framework, but are available from the corresponding author on reasonable request.

## Abbreviation

CBHC: Community Based Health Care
